# Biluo Qianyuan Formula Ameliorates Post-Traumatic Osteoarthritis by Suppressing FN1-Mediated Synovial Inflammation and Restoring Joint Homeostasis

**DOI:** 10.3390/ph19030500

**Published:** 2026-03-18

**Authors:** Yinqiu Wu, Guangran Hu, Shengzhe Zhang, Guilan Jin, Hua Dai

**Affiliations:** 1 School of Basic Medical Sciences & School of Public Health, Faculty of Medicine, Yangzhou University, Yangzhou 225001, China; 13852579987@163.com (Y.W.); 13837326996@163.com (G.H.);; 2 The Key Laboratory of the Jiangsu Higher Education Institutions for Nucleic Acid & Cell Fate Regulation, Yangzhou University, Yangzhou 225001, China; 3 School of Basic Medical Sciences, Nanjing University of Chinese Medicine, Nanjing 210023, China

**Keywords:** post-traumatic osteoarthritis, Biluo Qianyuan Formula, synovial inflammation, fibronectin 1, fibroblast-like synoviocytes, disease-modifying therapy

## Abstract

**Background**: Post-traumatic osteoarthritis (PTOA) lacks effective disease-modifying therapies that preserve joint structure while promoting tissue repair. This study aimed to evaluate the therapeutic efficacy and underlying mechanism of Biluo Qianyuan Formula (BLQYF), a standardized herbal formulation derived from clinical practice, as a potential disease-modifying alternative to celecoxib in a murine model of PTOA. **Methods**: A murine PTOA model was established and treated with BLQYF at different doses, with celecoxib serving as a pharmacological comparator. Safety was assessed by hepatic and renal toxicity analyses. Therapeutic effects were evaluated using micro-computed tomography (micro-CT) and histological staining. Network-based integrative analyses were conducted to identify key regulatory targets, followed by experimental validation in fibroblast-like synoviocytes. **Results**: BLQYF was well tolerated under the experimental conditions, with no detectable hepatic or renal toxicity at therapeutic doses. Micro-CT and histological analyses demonstrated that BLQYF dose-dependently mitigated subchondral bone deterioration, enhanced cartilage regeneration, and restored collagen deposition. At higher doses, BLQYF showed therapeutic efficacy comparable to celecoxib, with superior outcomes regarding cartilage reparation. Mechanistically, integrative analyses identified fibronectin 1 (FN1) as a central regulatory hub. Validation experiments confirmed that BLQYF suppressed FN1, MMP3, and TGF-β expression in fibroblast-like synoviocytes, thereby attenuating inflammation and extracellular matrix degradation. **Conclusions**: These findings support BLQYF as a promising disease-modifying therapeutic candidate for PTOA and highlight the fibroblast–FN1 axis as a novel pharmacological target for intervention.

## 1. Introduction

Post-traumatic osteoarthritis (PTOA) is a distinct subtype of osteoarthritis (OA) that develops following joint injury, such as intra-articular fracture, ligament rupture, or meniscal damage [1,2]. Unlike primary OA, which evolves slowly over decades, PTOA is characterized by a rapid onset and accelerated structural deterioration, often affecting younger and physically active individuals [3]. As the incidence of sports-related injuries and high-energy trauma continues to rise worldwide, PTOA has emerged as a growing public health concern with substantial long-term socioeconomic impact [4]. Despite this burden, PTOA remains clinically managed largely under the therapeutic framework of generalized OA, with limited consideration of its unique pathophysiological drivers [5,6].

Current treatment strategies for PTOA are primarily symptom-oriented. Non-steroidal anti-inflammatory drugs (NSAIDs), such as celecoxib, can alleviate pain and suppress acute inflammation but fail to prevent progressive cartilage loss or aberrant subchondral bone remodeling [7,8]. Moreover, prolonged NSAID use may interfere with tissue repair processes, underscoring the unmet need for disease-modifying osteoarthritis drugs (DMOADs) [9]. Efforts to develop DMOADs have been largely unsuccessful, in part because traditional approaches focus on single molecular targets or isolated tissues. Increasing evidence instead supports the view that PTOA is a whole-joint disease initiated by mechanical injury and sustained by complex interactions among cartilage, synovium, subchondral bone, and the immune system [10,11]. How to restore joint homeostasis at a systems level remains a fundamental challenge in PTOA therapy.

Synovial microenvironment remodeling has emerged as a critical pathological nexus linking acute injury to chronic joint degeneration. Mechanical overload and injury-induced inflammatory cues rapidly activate synovial fibroblasts, driving their transition toward pro-inflammatory and pro-fibrotic phenotypes [12,13,14]. These activated fibroblasts, in turn, amplify inflammatory signaling and promote pathological extracellular matrix (ECM) remodeling, establishing a self-sustaining degenerative cascade [15,16,17]. Among ECM-associated molecules, fibronectin 1 (FN1) has gained increasing attention as a key regulatory factor in OA pathogenesis [16]. FN1 is a major ECM glycoprotein involved in tissue repair; however, its aberrant overexpression and proteolytic fragments can act as damage-associated molecular patterns (DAMPs), activating inflammatory pathways and inducing matrix metalloproteinase expression, thereby accelerating cartilage degradation [18]. In parallel, dysregulated transforming growth factor-β (TGF-β) signaling interacts with FN1-rich matrices to promote synovial fibrosis and maladaptive joint remodeling [19,20]. Despite growing recognition of the fibroblast–FN1 axis as a central driver of joint degeneration, effective therapeutic strategies capable of systemically modulating this pathological network are still lacking.

In this context, traditional multi-component therapies offer a conceptually distinct approach to complex degenerative diseases such as PTOA. Unlike single-target drugs, multi-component formulations have the potential to act on multiple signaling pathways and cell types simultaneously, thereby addressing the systems-level imbalance characteristic of whole-joint disorders [21,22]. *Biluo Qianyuan Formula* (BLQYF) is a standardized herbal formulation developed by the National Experimental Teaching Demonstration Center of Nanjing University of Chinese Medicine, based on long-term clinical practice and subsequently refined and standardized by the research group led by Professor Guilan Jin. The formulation consists of nine medicinal components combined in defined relative proportions, with Astragalus membranaceus and Ephedra sinica as the dominant constituents, complemented by herbs traditionally associated with anti-inflammatory regulation, fibrosis modulation, and bone metabolism [23,24,25,26].

Emerging pharmacological evidence supports the biological plausibility of this multi-target strategy. Astragalus-derived polysaccharides and saponins have been shown to modulate innate immune responses and suppress pro-inflammatory cytokine production [27]. Compounds associated with Ephedra [28] and Ligusticum chuanxiong [29,30] have demonstrated inhibitory effects on NF-κB signaling and microcirculatory improvement. Additional components, including Clematis chinensis and Psoralea corylifolia, contain bioactive molecules implicated in anti-fibrotic regulation and bone metabolic pathways, while Achyranthes bidentata [31,32], Poria cocos [33], and Glycyrrhiza uralensis [34,35] have been linked to immune homeostasis and oxidative stress modulation. Although the molecular actions of individual constituents have been partially characterized, how such components act cooperatively within a defined formulation to regulate key pathological nodes in PTOA remains largely unexplored.

Here, we sought to investigate whether a multi-component therapeutic strategy could systemically modulate PTOA progression by targeting core pathological axes at the cellular and molecular levels. Specifically, this study focuses on the hypothesis that fibroblast-centered FN1 dysregulation represents a critical, therapeutically actionable node in PTOA. By integrating systems pharmacology, single-cell transcriptomics, and multi-scale experimental analyses, we aim to delineate the molecular mechanisms through which BLQYF influences joint inflammation, matrix remodeling, and tissue homeostasis. Addressing these questions may not only advance mechanistic understanding of PTOA pathogenesis but also provide a rational framework for the development of multi-target disease-modifying therapies for post-traumatic joint degeneration.

## 2. Results

### 2.1. BLQYF Attenuates Bone and Cartilage Structural Damage in PTOA Mouse Knees

To evaluate systemic safety, hepatic and renal biochemical parameters were assessed. In addition to AST, ALT, creatinine (Cr), and blood urea nitrogen (BUN), serum alkaline phosphatase (ALP), γ-glutamyl transferase (GGT), and total bilirubin (TBil) were measured. All values remained within physiological ranges and did not differ significantly among groups (Supplementary Figure S1A–L). Consistently, hematoxylin and eosin (H&E) staining of liver and kidney tissues showed preserved architecture without overt structural abnormalities (Supplementary Figure S1M), suggesting no detectable hepatic or renal toxicity under the present experimental conditions.

**Figure 1:**
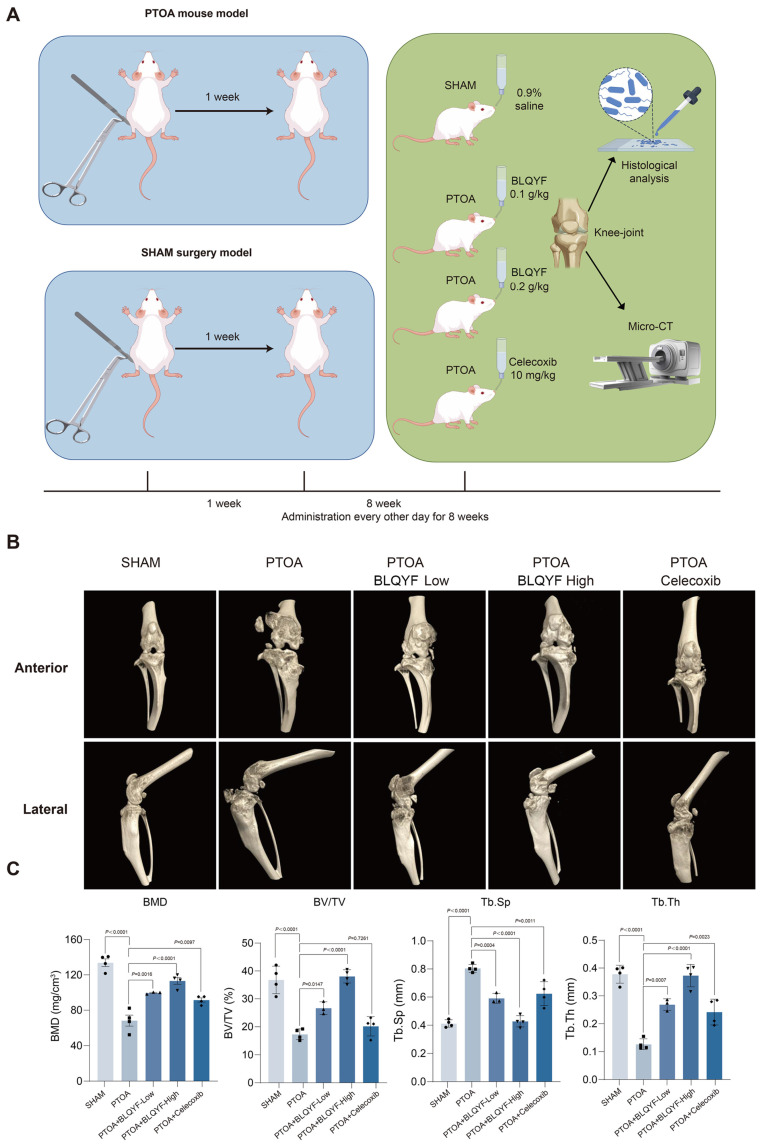
Micro-CT assessment of knee joint structure in a PTOA mouse model. (**A**) Schematic of the experimental design for inducing PTOA in mice (surgical injury vs. sham surgery) and the subsequent treatment regimen with BLQYF (low or high dose) or a positive control drug (celecoxib) administered over 8 weeks. (**B**) Representative micro-computed tomography (micro-CT) 3D reconstructions of the knee joint subchondral bone from sham-operated mice, untreated PTOA model mice, and PTOA model mice treated with low-dose BLQYF, high-dose BLQYF, or celecoxib. (**C**) Quantitative micro-CT analysis of subchondral bone microarchitecture, including bone mineral density (BMD), bone volume fraction (BV/TV), trabecular thickness (Tb.Th), and trabecular separation (Tb.Sp). Data are shown as mean ± SEM. The data were analyzed by two-way ANOVA or one-way ANOVA, followed by Tukey’s post hoc test for multiple comparisons.

The therapeutic effects of BLQYF were subsequently assessed in a post-traumatic osteoarthritis (PTOA) mouse model. Micro-computed tomography (micro-CT) imaging and three-dimensional reconstruction revealed pronounced joint structural alterations in PTOA mice, including meniscal damage, focal cartilage surface irregularities, and reduced chondrocyte density. BLQYF treatment was associated with a dose-dependent attenuation of these pathological features, with higher doses showing improved preservation of articular surface integrity and cartilage thickness relative to untreated PTOA mice (Figure 1A,B).

Consistent with these observations, quantitative analysis of subchondral bone parameters demonstrated that BLQYF treatment partially reversed PTOA-associated reductions in bone volume fraction (BV/TV) and bone mineral density (BMD), while also decreasing trabecular separation (Tb.Sp) (Figure 1C). The magnitude of these changes was comparable to that observed in the celecoxib-treated group.

Together, these results suggest that BLQYF is well tolerated in vivo and is associated with improvements in joint structural parameters and subchondral bone microarchitecture in PTOA mice.

### 2.2. The Effect of BLQYF Treatment on Knee Cartilage Regeneration and Repair in PTOA Mice

To evaluate the effects of BLQYF on joint structure in PTOA mice, histological analyses were performed after 8 weeks of treatment.

**Figure 2:**
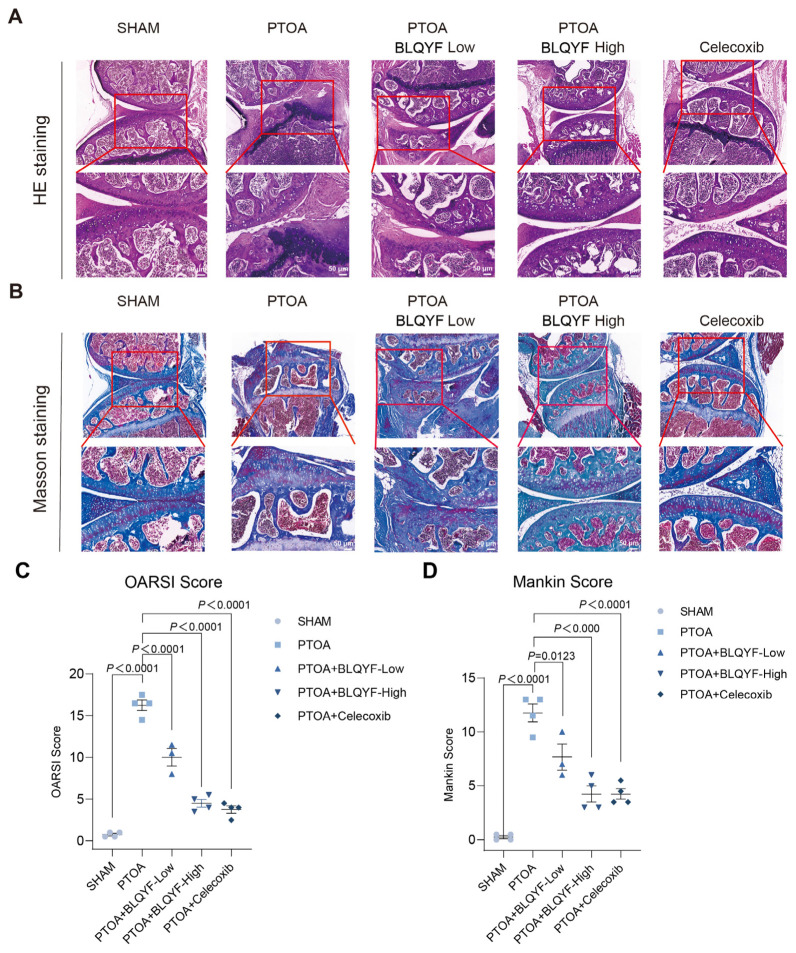
Histological assessment and cartilage degeneration scoring. (**A**) Representative H&E staining. Upper panels: 10× images showing overall joint architecture; lower panels: 20× images highlighting structural details. (**B**) Representative Masson’s trichrome staining. Upper panels: 10× images; lower panels: 20× images. (**C**) Osteoarthritis Research Society International (OARSI) scores quantifying the severity of cartilage degeneration. (**D**) Mankin scores evaluating histopathological cartilage damage. Data are presented as mean ± SEM. Statistical significance was determined using appropriate statistical tests.

H&E staining revealed marked whole-joint alterations (Figure 2A). The SHAM group displayed intact cartilage, preserved subchondral bone architecture, and normal synovial morphology. In contrast, PTOA mice showed severe cartilage erosion, chondrocyte loss, subchondral bone remodeling with irregular trabeculae, and synovial thickening.

BLQYF treatment attenuated these pathological changes in a dose-dependent manner. The high-dose group exhibited improved cartilage surface integrity, more organized trabecular structure, and reduced synovial hyperplasia, comparable to celecoxib.

Masson’s trichrome staining further demonstrated improved collagen distribution in BLQYF-treated groups compared with the PTOA group (Figure 2B).

Consistently, OARSI and modified Mankin scores confirmed significant cartilage degeneration in PTOA mice, which was markedly reduced following BLQYF treatment (Figure 2C,D).

### 2.3. UPLC–MS/MS-Based Chemical Characterization of BLQYF

**Figure 3:**
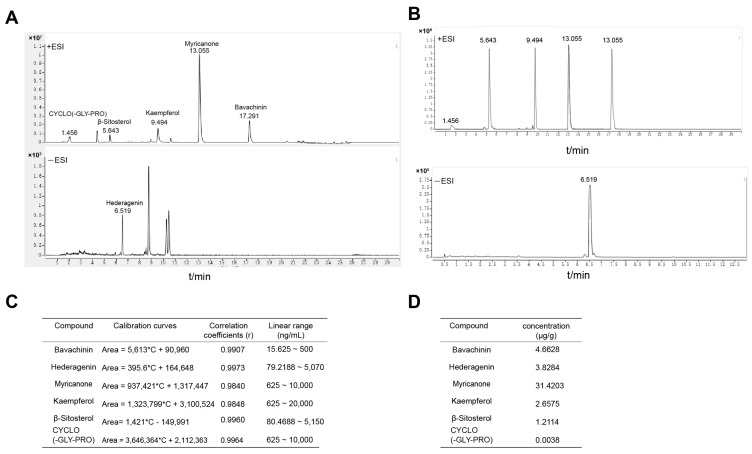
Quantitative analysis of representative active components in BLQYF. (**A**) The UPLC chromatogram of the BLQYF sample. (**B**) The UPLC chromatogram of a mixed reference standard containing six active components. (**C**) Calibration curves for individual compounds were generated for quantitative analysis using high-performance liquid chromatography (HPLC). Linear regression analysis was performed across a series of standard concentrations to determine the correlation coefficients (r) and linear ranges (ng mL^−1^) for each compound; detailed calibration parameters are provided in the table. (**D**) Limits of detection (LODs; μg g^−1^) for each compound were defined as the lowest concentration reliably detected under the established calibration conditions. Analytical conditions for LOD determination were identical to those used for quantitative analysis.

To define the chemical basis underlying BLQYF activity, we profiled BLQYF extracts by UPLC–MS/MS. The UPLC–MS/MS analysis revealed multiple well-resolved peaks, and key retention times were consistent with those of a mixed reference containing six authentic standards (Figure 3A,B). Peak assignment confirmed the presence of bavachinin, hederagenin, myricanone, kaempferol, β-sitosterol, and cyclo(Gly–Pro) in BLQYF.

For quantification, calibration curves for each analyte showed excellent linearity (Supplementary Figure S2A–F; R^2^ > 0.99). Among the quantified constituents, myricanone was the most abundant (3.4567 μg g^−1^), followed by cyclo(Gly–Pro) (2.6575 μg g^−1^) and a third compound (1.2114 μg g^−1^), whereas ursolic acid was detected at a markedly lower level (0.0038 μg g^−1^) (Figure 3C,D). Collectively, these data establish a reproducible UPLC–MS/MS chemical fingerprint for BLQYF and support the presence of multiple bioactive constituents.

### 2.4. Network Pharmacology and Target Pathway Analysis of BLQYF in PTOA

**Figure 4:**
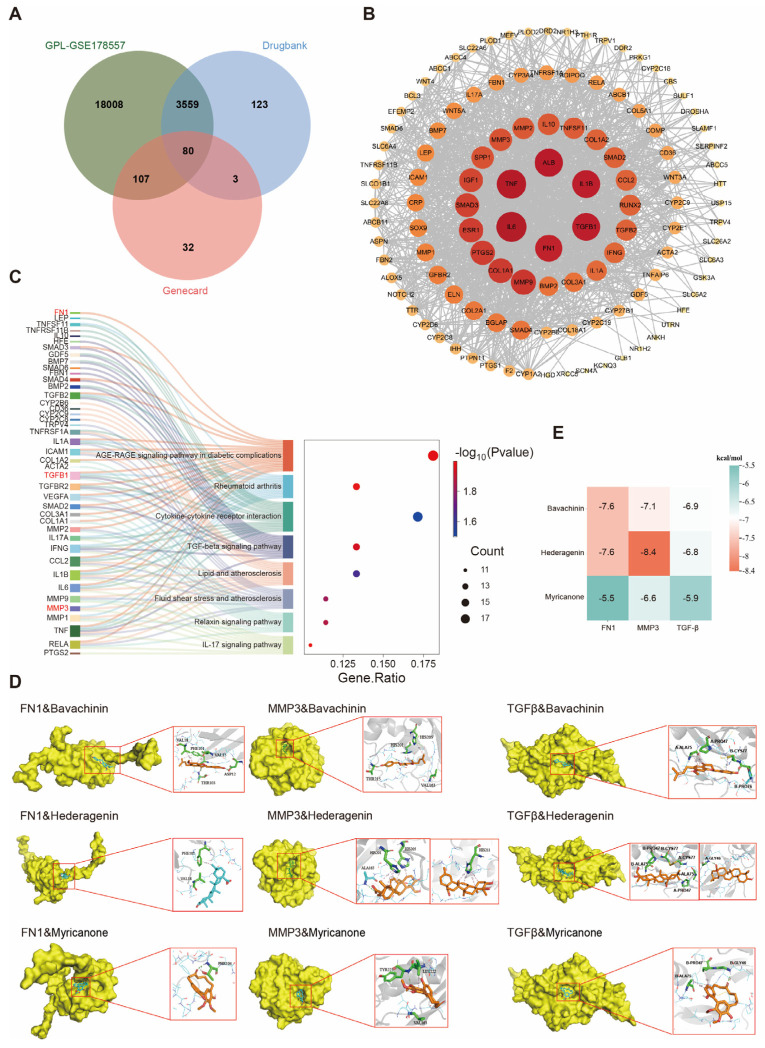
Integrated network pharmacology and molecular docking analysis of candidate compounds and targets. (**A**) A Venn diagram illustrates the intersection of differentially expressed genes from the GSE178557 dataset with disease-related genes from GeneCards and drug-target genes from DrugBank. (**B**) The PPI network of overlapping targets was generated to visualize functional associations. Central nodes (red) indicate core hub genes identified based on degree centrality and connectivity. (**C**) A Sankey diagram correlates specific target genes (**left**) with their respective enriched KEGG biological pathways (**right**). Connectivity indicates involvement in signaling cascades such as the AGE-RAGE, TGF-beta, and IL-17 signaling pathways. (**D**) Representative 3D docking poses demonstrate the binding orientations and intermolecular interactions between active compounds (bavachinin, hederagenin, and myricanone) and primary protein targets (FN1, MMP3, and TGF-β). (**E**) A heatmap displays the molecular docking scores (binding energy, kcal/mol) for the interaction between candidate compounds and hub targets. Color intensity and numerical values represent the predicted binding stability.

To systematically explore the molecular mechanisms by which BLQYF may exert therapeutic effects in PTOA, a network pharmacology approach was employed to identify putative targets and signaling pathways. Candidate bioactive constituents of BLQYF were retrieved from the Traditional Chinese Medicine Systems Pharmacology Database (TCMSP) using oral bioavailability ≥ 30% and drug-likeness ≥ 0.18 as screening criteria, and their potential targets were predicted using SwissTargetPrediction. In parallel, PTOA -associated targets were collected from GeneCards and GEO databases. Intersection analysis of these datasets yielded a set of common BLQYF–PTOA targets (Figure 4A), suggesting a potential molecular basis for BLQYF intervention in PTOA.

To further characterize the interactions among these shared targets, a protein–protein interaction (PPI) network was constructed (Figure 4B). Topological analysis identified fibronectin 1 (FN1) as a prominent hub gene (degree = 60), positioned at the core of the network together with classical inflammatory mediators such as IL-6 and TNF. Notably, FN1 exhibited direct interactions with IL-6, TGF-β, matrix metalloproteinase-3 (MMP3), and other key regulators, indicating extensive connectivity linking inflammatory signaling with extracellular matrix (ECM) organization. This central role of FN1 is consistent with accumulating evidence that FN1 is a critical regulator in osteoarthritis pathogenesis, and that fibronectin fragments actively promote inflammatory responses and cartilage degeneration in PTOA models [36].

Consistent with the multi-component nature of BLQYF, the compound–target–disease network (Supplementary Figure S3A) revealed that multiple active compounds were connected to numerous PTOA-related targets. Several compounds displayed relatively high degree values, indicating broad target engagement and suggesting that they may represent key bioactive constituents contributing to the therapeutic effects of BLQYF. To gain functional insight into these shared targets, Gene Ontology (GO) enrichment analysis was performed, revealing significant enrichment in biological processes associated with inflammatory responses, immune regulation, and cellular stress. Complementary pathway enrichment analysis demonstrated that the identified targets were significantly enriched in several inflammation- and metabolism-related pathways, including the AGE–RAGE signaling pathway in diabetic complications, cytokine–cytokine receptor interaction, rheumatoid arthritis, and the TGF-β signaling pathway (Supplementary Figure S3B). These pathways are closely associated with immune regulation, inflammatory responses, and extracellular matrix remodeling, suggesting their potential involvement in synovial inflammation and cartilage homeostasis during the progression of post-traumatic osteoarthritis (PTOA).

To integrate the relationships between core targets and enriched pathways, a Sankey diagram was generated (Figure 4C). This visualization revealed that FN1 is involved in multiple inflammation-, immunity-, and ECM-related pathways, highlighting its widespread participation across diverse PTOA-associated signaling networks. Collectively, these network pharmacology results support a multi-target, multi-pathway mechanism by which BLQYF may modulate inflammatory signaling, immune responses, and extracellular matrix remodeling through the regulation of central nodes such as FN1. This systems-level perspective provides a mechanistic framework that rationalizes the therapeutic potential of BLQYF and supports the subsequent in vivo experimental observations.

To further validate potential compound–target interactions suggested by the network analysis, molecular docking simulations were conducted between six representative BLQYF compounds and three key target proteins—FN1, MMP3, and TGF-β (Figure 4D,E). The docking results showed that bavachinin, hederagenin, and myricanone exhibited the lowest binding free energies among the tested compounds, indicating stronger binding affinities toward these target proteins. These findings suggest that these compounds may play dominant roles in mediating the regulatory effects of BLQYF on PTOA-related signaling pathways.

### 2.5. Single-Cell Transcriptomic Analysis Identifies Fibroblast-Associated FN1 Expression Modules in Osteoarthritic Tissues

**Figure 5:**
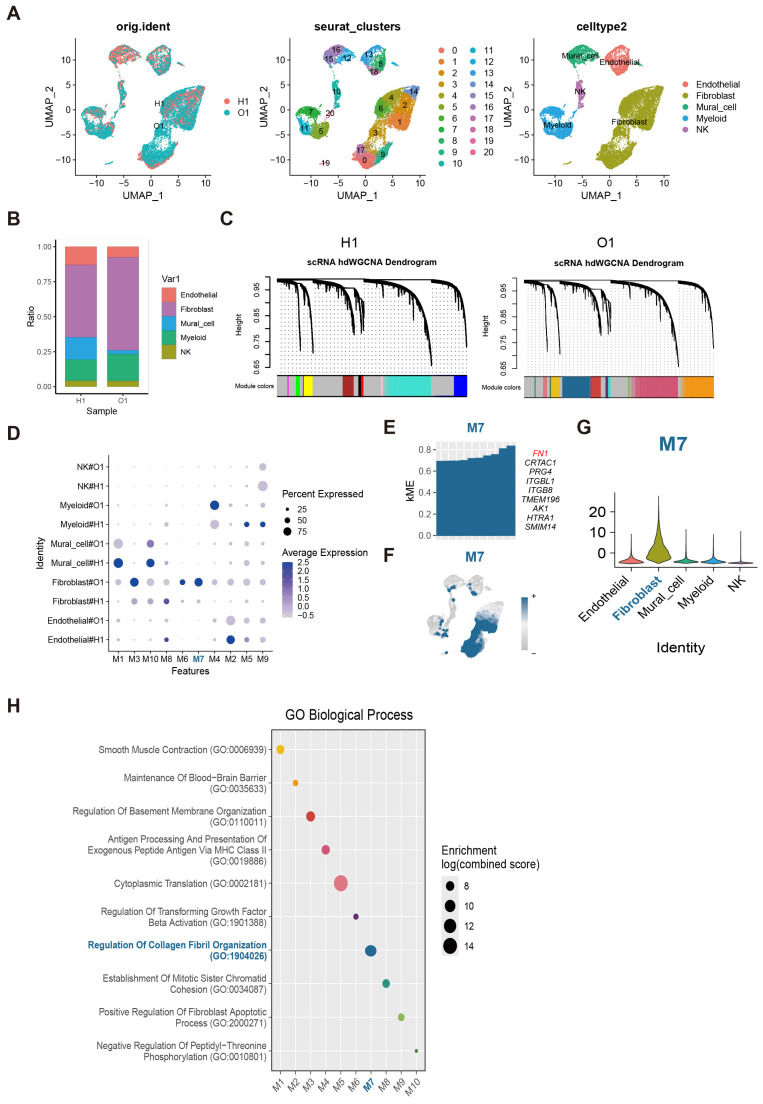
Single-cell transcriptional landscape and gene co-expression network analysis of osteoarthritic tissues. (**A**) Uniform manifold approximation and projection (UMAP) visualization of single cells derived from the GSE216651 dataset. The panels display cells colored by sample origin (H1 and O1, left), unsupervised Seurat clusters (middle), and annotated major cell types (right). (**B**) Stacked bar chart illustrating the relative cellular composition of the five identified cell types across samples H1 and O1. (**C**) High-dimensional weighted gene co-expression network analysis (hdWGCNA) dendrograms showing the hierarchical clustering of genes into co-expression modules for H1 and O1 datasets. The color bars at the bottom represent the distinct gene modules identified. (**D**) Dot plot summarizing the expression of identified gene modules (M1–M10) across different cell types and conditions. The size of the dot represents the percentage of cells expressing the module genes, and color intensity indicates the average expression level. (**E**) Intramodular connectivity (kME) was calculated to assess gene importance within Module 7 (M7). The top-ranking genes were prioritized and visualized via a bar plot, with representative hub genes indicated. (**F**) M7 module scores were projected onto the UMAP embedding using feature plots to visualize the distribution of module-specific activity across the single-cell landscape. (**G**) The distribution of M7 module scores across five major cell lineages was evaluated and compared using violin plots. (**H**) Bubble plot of Gene Ontology (GO) biological process enrichment analysis for the identified modules. The *x*-axis represents the modules, and the *y*-axis lists representative GO terms. The size of the bubbles corresponds to the enrichment significance (log (combined score)). The different colors are used to distinguish different GO biological processes.

To delineate cell-type-specific transcriptional programs in osteoarthritic (OA) tissues, single-cell RNA sequencing data from the GEO dataset GSE216651 were analyzed. Following quality control and normalization, uniform manifold approximation and projection (UMAP) revealed well-resolved cellular structures with substantial overlap between samples from healthy (H1) and osteoarthritic (O1) donors, indicating minimal batch effects (Figure 5A). Unsupervised clustering identified 20 distinct clusters, which were annotated based on established marker genes, encompassing endothelial cells, fibroblasts, mural cells, myeloid cells, and natural killer (NK) cells (Figure 5A).

Comparative analysis of cellular composition showed shifts in the relative abundance of major cell populations between H1 and O1 samples, although all cell types were present in both conditions (Figure 5B). Canonical marker gene expression further validated the robustness of cell-type annotation (Supplementary Figure S4A), with markers such as *PECAM1* and *CDH5* delineating endothelial cells, *COL1A1* and *COL1A2* identifying fibroblasts, and *RGS5* and *MCAM* marking mural cells.

To investigate coordinated transcriptional programs beyond individual genes, single-cell weighted gene co-expression network analysis (scRNA-seq WGCNA) was performed separately for H1 and O1 samples. This analysis grouped co-expressed genes into ten modules (M1–M10), each displaying distinct cell-type-associated expression patterns (Figure 5C,D and Supplementary Figure S4B–D). Several modules exhibited preferential enrichment in fibroblasts, among which module M7 showed the most pronounced fibroblast specificity (Figure 5E). Core genes within this module included *FN1*, *PRG4*, and *ITGB1*, whose expression was largely restricted to fibroblasts, as demonstrated by violin plot analysis (Figure 5F,G and Supplementary Figure S4E).

The fibroblast-specific enrichment of *FN1* aligns with its identification as a central hub gene in the BLQYF–OA network, reflecting its dual role as a disease marker and a mediator of inflammatory signaling and matrix catabolism. This convergence of single-cell evidence and network pharmacology suggests that BLQYF targets *FN1* to modulate fibroblast-driven extracellular matrix remodeling, potentially reconciling its complex, context-dependent functions in joint homeostasis.

Consistent with these findings, functional enrichment analysis of genes within fibroblast-enriched modules revealed significant overrepresentation of biological processes related to basement membrane organization, regulation of collagen fibril organization, smooth muscle contraction, and transforming growth factor-β (TGF-β) activation (Figure 5H). These pathways are closely linked to fibroblast activation, ECM remodeling, and fibrotic responses, which are increasingly recognized as key contributors to OA-associated tissue degeneration and maladaptive repair. Together, these data highlight fibroblast-centered transcriptional programs, characterized by elevated *FN1* expression, as integral components of OA pathophysiology and as potential cellular targets underlying the therapeutic effects of BLQYF.

### 2.6. BLQYF Suppresses Inflammatory Responses by Targeting FN1 in FLSs Derived from Patients with Post-Traumatic Osteoarthritis

**Figure 6:**
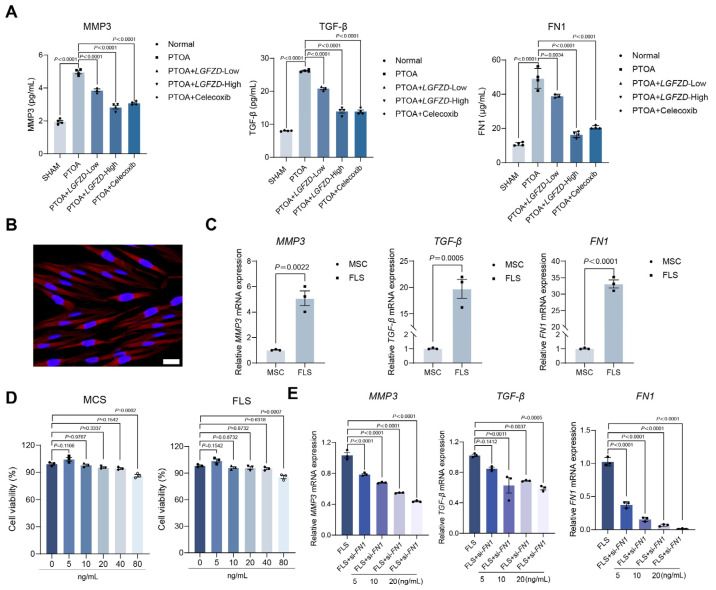
BLQYF attenuates systemic inflammation and FN1-associated responses in PTOA. (**A**) Serum levels of MMP3, TGF-β, and FN1 in sham, PTOA, and PTOA mice treated with BLQYF or celecoxib. (**B**) Representative morphology and vimentin immunofluorescence staining of primary fibroblast-like synoviocytes (FLSs) isolated from synovial tissues of patients with post-traumatic osteoarthritis. Nuclei were counterstained with DAPI. Scale bar = 20 μm. (**C**) qRT–PCR analysis of *MMP3*, *TGF-β*, and *FN1* mRNA expression in FLSs from PTOA patients and mesenchymal stem cells (MSCs) from non-osteoarthritic hip arthroplasty donors. (**D**) CCK-8 assay showing MSC viability following BLQYF treatment (0–80 ng/mL, 24 h). (**E**) qRT–PCR analysis of *MMP3*, *TGF-β*, and *FN1* expression in FLSs following FN1 knockdown and BLQYF treatment (5, 10, 20 ng/mL, 24 h). Data were presented as mean ± SEM. Statistical significance was determined by one-way ANOVA with appropriate post hoc tests. *p* < 0.05, *p* < 0.01, *p* < 0.001.

In a murine model of PTOA, BLQYF administration significantly suppressed the systemic surge of extracellular matrix-associated and inflammatory mediators. Serum levels of MMP3, TGF-β, and fibronectin 1 (FN1), which were markedly elevated following joint injury, were reduced in a dose-dependent manner, with the high-dose BLQYF cohort achieving biomarker profiles comparable to those of celecoxib-treated animals (Figure 6A). These findings suggest that BLQYF exerts COX-2-comparable efficacy in restraining joint injury-induced systemic inflammation and ECM remodeling. Given the increasing recognition of ECM components as immunomodulatory scaffolds, the reversal of FN1 elevation is particularly notable and raises the possibility that BLQYF may influence the matrix–immune cell cross-talk central to PTOA progression.

To identify the cellular source of this inflammatory signature, we analyzed fibroblast-like synoviocytes (FLSs) isolated from synovial tissue of PTOA patients. These cells exhibited classical mesenchymal morphology and vimentin positivity (Figure 6B), consistent with their activated fibroblastic identity. Comparative gene expression profiling demonstrated a marked upregulation of *MMP3*, *TGF-β*, and *FN1* in FLSs relative to mesenchymal stem cells (MSCs) derived from non-arthritic donors (Figure 6C), supporting their role as key effector cells in sustaining inflammation and matrix degradation in PTOA. These findings are in line with prior studies identifying synovial fibroblasts as dominant amplifiers of chronic inflammation in both rheumatoid and osteoarthritic contexts [14,17,37].

Assessment of BLQYF cytocompatibility in MSCs confirmed minimal toxicity at concentrations up to 40 ng/mL, with a significant decline in viability observed at 80 ng/mL (Figure 6D). This concentration range thus delineates a therapeutic window for mechanistic and translational interrogation.

To probe the mechanistic dependence of BLQYF on FN1 signaling, we employed siRNA-mediated FN1 silencing in FLSs. As anticipated, FN1 knockdown reduced basal expression of MMP3 and TGF-β. Intriguingly, BLQYF treatment led to further, dose-dependent suppression of these mediators even in the context of FN1 depletion (Figure 6E). This additive effect suggests that BLQYF may modulate downstream transcriptional programs or parallel signaling nodes beyond FN1 abundance per se. While FN1 was predicted as a central node in network pharmacology models, these results imply that BLQYF may also target convergent inflammatory axes or modulate FN1 post-transcriptional activity.

Collectively, these data indicate that BLQYF orchestrates PTOA pathogenesis by modulating fibroblast-mediated inflammation and ECM dynamics through the FN1 axis, though its precise molecular interactome remains to be fully elucidated. Future translational optimization will necessitate structural characterization of its binding partners and time-resolved single-cell profiling to define its therapeutic impact within the evolving joint microenvironment.

## 3. Discussion

Post-traumatic osteoarthritis (PTOA) remains a major unmet clinical challenge, largely due to a fundamental limitation of current standard-of-care therapies [38,39]. Non-steroidal anti-inflammatory drugs (NSAIDs), such as celecoxib, are effective in alleviating pain and suppressing inflammation, yet their benefits are predominantly symptomatic and do not prevent progressive joint structural deterioration [40,41]. Moreover, prolonged suppression of inflammatory signaling may interfere with endogenous tissue repair processes. Addressing this long-standing therapeutic dilemma was the central objective of the present study.

Here, we demonstrate that BLQYF exerts significant anti-inflammatory effects and modulates joint-related inflammatory mediators comparable to celecoxib, while offering superior protection against joint structural degeneration. Notably, BLQYF preserved cartilage integrity and significantly improved subchondral bone microarchitecture, as evidenced by increased bone volume fraction (BV/TV) and reduced trabecular separation (Tb.Sp) in micro-CT analyses. Given the critical role of subchondral bone in distributing mechanical load and mediating biochemical interactions between bone and cartilage, these findings underscore the importance of targeting the osteochondral unit as a whole, rather than focusing solely on cartilage. Collectively, our results support the emerging understanding that osteoarthritis is not merely a cartilage-centric disorder, but rather a multifaceted failure of the whole-joint organ, involving cartilage, synovium, subchondral bone, and immune components. By orchestrating a coordinated multi-component action, BLQYF shifts the therapeutic focus from short-term symptom management to the restoration of intra-articular homeostasis.

A key mechanistic insight of this study arises from the integration of single-cell RNA sequencing (scRNA-seq) with network pharmacology, which facilitated the identification and validation of a fibroblast–fibronectin 1 (FN1)–TGF-β axis as a critical driver of PTOA progression. While synovitis has traditionally been regarded as a secondary response to cartilage damage, our single-cell analyses reveal that synovial fibroblast-like synoviocytes (FLSs) undergo disease-specific transcriptional reprogramming and become a dominant source of FN1 and profibrotic extracellular matrix components. These findings suggest that synovial fibroblasts may play an active role in disease initiation and progression, rather than merely serving as passive responders to structural injury.

Mechanistically, the sustained pathological activation of TGF-β signaling promotes the fibroblast-to-myofibroblast transition (FMT), resulting in the excessive deposition of FN1- and collagen-rich extracellular matrix. Beyond inducing synovial fibrosis and joint stiffness, the FN1-enriched matrix acts as a damage-associated molecular pattern (DAMP), amplifying inflammatory signaling through integrin-mediated pathways and establishing a self-reinforcing pathological loop that continuously compromises adjacent cartilage tissue. BLQYF effectively attenuated the activation of this fibrotic cascade, functionally disrupting the transmission of destructive signals from the synovium to the cartilage. These findings have significant translational implications, suggesting that targeting synovial fibrosis may be a particularly effective strategy for early-to-mid-stage PTOA, potentially outperforming current approaches that focus exclusively on chondrocytes.

The therapeutic efficacy of BLQYF does not rely on a single molecular target but emerges from the coordinated actions of multiple bioactive constituents that collectively reprogram the joint pathological network. Through UPLC–MS/MS profiling coupled with molecular docking and network analyses, we elucidated the systems pharmacological basis of BLQYF across three interdependent dimensions. Bavachinin [42,43], derived from *Psoralea corylifolia*, was identified as a natural pan-PPAR agonist capable of improving local metabolic homeostasis, enhancing mitochondrial function, and reducing oxidative stress in the hypoxic and inflammatory PTOA microenvironment. This metabolic modulation provides an essential energetic foundation for tissue repair. Myricanone [44,45], originating from *Ligusticum chuanxiong*, selectively suppressed inflammatory cascades by targeting upstream kinases such as AKT1 and SRC, thereby attenuating NF-κB-associated signaling and limiting M1 polarization of synovial macrophages without inducing broad immunosuppression [46,47]. Hederagenin [48,49], a key component of *Clematis chinensis*, directly interfered with TGF-β-induced FMT through modulation of Keap1–Nrf2 and Ras/JNK signaling, resulting in reduced pathological FN1 deposition and synovial hyperplasia [50]. Collectively, these pleiotropic effects establish an integrated regulatory landscape across metabolic, immune, and ECM dimensions; however, further in vivo deconvolution—benchmarking the holistic BLQYF extract against its primary bioactive monomers—is warranted to substantiate the discrete pharmacodynamic contributions and synergistic interactions underlying this multi-target intervention.

Standardization and reproducibility are critical considerations in studies involving multi-component botanical formulations. To ensure chemical consistency, BLQYF was characterized using UPLC–MS/MS profiling, and major bioactive constituents were consistently detected across experimental batches. Quality control procedures were implemented to maintain compositional stability and batch-to-batch reproducibility. Several principal constituents of BLQYF, including bavachinin, myricanone, and hederagenin, have been individually reported to exert anti-inflammatory, metabolic-regulatory, or anti-fibrotic activities. However, these compounds act on partially overlapping yet distinct molecular pathways, including PPAR signaling, NF-κB–associated inflammatory cascades, and TGF-β–mediated fibrotic remodeling. Our integrative systems-level analyses suggest that the therapeutic efficacy of BLQYF is unlikely to depend on a single dominant component, but rather arises from coordinated modulation of interconnected pathological networks governing metabolism, immune regulation, and extracellular matrix homeostasis. Although further studies dissecting the relative contribution of individual constituents would be informative, the present findings support the concept that multi-component formulations may achieve therapeutic benefit through network-level and potentially synergistic regulation of complex joint pathology.

Notwithstanding the strengths of this study, several aspects warrant further consideration. Although no overt biochemical or histopathological abnormalities were observed, the safety conclusions are based on a limited set of markers. The biochemical parameters and H&E staining revealed no significant abnormalities, but these markers may not detect subtle or chronic toxicity. Additionally, organ weight assessment was not included, which is a limitation relative to ICH and OECD toxicology guidelines. Future studies should include more comprehensive toxicological evaluations to clarify the full safety profile of BLQYF, including long-term monitoring and detailed assessments of TGF-β signaling modulation. TGF-β signaling is known to exert a context-dependent dual role in joint biology, supporting tissue homeostasis under physiological conditions while promoting fibrotic remodeling upon sustained or excessive activation [39,40,41,42]. Although the present data suggest that BLQYF attenuates pathological TGF-β signaling, the precise mechanisms by which this multi-component formula achieves functional pathway rebalancing, rather than nonspecific inhibition, remain to be fully elucidated. In particular, whether BLQYF exerts cell type-specific, state-dependent, or temporally restricted effects on distinct nodes of the TGF-β signaling cascade requires further investigation through higher-resolution spatiotemporal analyses across different joint compartments. Although no overt biochemical or histopathological abnormalities were observed, organ weight assessment was not included, representing a limitation relative to ICH and OECD toxicology recommendations. This will be addressed in future studies. Additionally, while the 8-week observation period demonstrated robust structural preservation, it may not fully capture the protracted and insidious progression of PTOA-associated fibrosis. Future longitudinal studies extending this timeframe are necessary to evaluate the long-term impact of BLQYF on chronic joint remodeling.

Despite these considerations, this study offers a broadly applicable conceptual framework for advancing the scientific modernization of traditional medicine. Recent developments in single-cell and spatial transcriptomics, together with cell–cell communication inference, enable in situ interrogation of how multi-component interventions remodel complex tissue microenvironments, thereby providing a molecular-level reinterpretation of traditional therapeutic principles. Furthermore, the identification of candidate biomarkers, including circulating FN1, TGF-β pathway activity, and disease-associated fibroblast-like synoviocyte (FLS) subpopulations, supports the development of biomarker-informed patient stratification strategies. Such approaches may facilitate a shift from empirically guided treatment toward a more precise and mechanism-oriented paradigm of traditional medicine. In parallel, the core bioactive constituents delineated in this study provide a rational foundation for the development of standardized, quality-controlled botanical drugs with increasingly well-defined pharmacological actions.

In summary, this work positions BLQYF as a promising disease-modifying candidate for post-traumatic osteoarthritis by reframing therapeutic intervention from cartilage-centric protection toward the restoration of whole-joint homeostasis. Through the integration of single-cell transcriptomics, systems pharmacology, and experimental validation, we identify a previously underappreciated fibroblast-driven fibrotic program and demonstrate the feasibility of system-level therapeutic modulation in complex joint diseases. More broadly, these findings exemplify how traditional multi-component therapies can be rigorously interrogated within modern biological frameworks, thereby advancing osteoarthritis research while contributing to the ongoing scientific modernization of traditional medicine.

## 4. Materials and Methods

### 4.1. Preparation of Biluo Qianyuan Formula

The Biluo Qianyuan Formula is composed of Ephedra (Ma Huang, minister herb), Astragalus root (Huang Qi, monarch herb), Chuanxiong rhizome (Chuan Xiong, assistant herb), Achyranthes root (Niu Xi, assistant/courier herb), Psoralea fruit (Bu Gu Zhi, minister herb), Poria (Fu Ling, assistant/courier herb), Clematis root (Wei Ling Xian, minister herb), Centipede (Wu Gong, assistant herb) and Licorice root (Gan Cao, assistant/courier herb). All botanical and zoological components were procured from Suzhou Tianling Pharmaceutical Co., Ltd. (Suzhou, China) and authenticated by Yangzhou Hospital of Traditional Chinese Medicine (Yangzhou, China), with verifiable provenance. The traditional prescription involves using the following herbs: Ma Huang, Huang Qi, Chuan Xiong, Niu Xi, Bu Gu Zhi, Fu Ling, Wei Ling Xian, Wu Gong, and Gan Cao. These herbs are soaked in ten-times their volume of distilled water for 30 min. After soaking, the mixture is decocted for one hour, filtered, and then decocted again for another 30 min. The combined decoctions are concentrated under reduced pressure using a rotary evaporator to achieve a final extract concentration of 1.0 g/mL of crude drug. This extract is stored at 4 °C and must be used within seven days. Before use, the decoction is warmed to room temperature.

BLQYF Working Solution for Cell Experiments: For cell culture experiments, the BLQYF working solution was prepared by dissolving the extract in phosphate-buffered saline (PBS) to obtain a final concentration of 1 mg/mL. The solution was freshly prepared for each experiment and used within 24 h to ensure consistency and avoid degradation.

### 4.2. Establishment of the PTOA (Post-Traumatic Osteoarthritis) Mouse Model

#### PTOA Mouse Modeling

Eight-week-old Balb/c mice (22 ± 2 g, SPF grade, from Yangzhou, China; production license SCXK [Su] 2024-0010) were anesthetized via paracentral injection of a 1% pentobarbital sodium solution (50 mg/kg). Post-anesthesia, the mice were placed supine, with limbs secured using tape. The fur around the right knee joint was shaved. The left index finger lifted the knee joint, while the right thumb pressed the ankle to flex the leg. A 3 mm incision was made along the inner edge of the patella, parallel to the patellar ligament. The medial joint capsule was cut to expose the medial meniscus of the tibial plateau. Microsurgical scissors were used to transect the medial meniscus (MMTL), destabilizing it. The wound was rinsed with saline, bleeding was controlled, and the incision was sutured and disinfected. The left knee joint was left intact. Mice in the SHAM group only underwent a skin incision and suturing. Post-operation, the mice were observed for 7 days. If no abnormal reactions occurred, drug administration began after a 1-day interval, continuing for 8 weeks.

The experimental mice were randomly allocated into four groups, each consisting of five mice: (1) a SHAM group, which underwent sham surgery and received normal saline; (2) a low-dose BLQYF group, receiving 0.1 g/kg of BLQYF every other day for 8 weeks; (3) a high-dose BLQYF group, receiving 0.2 g/kg of BLQYF every other day for 8 weeks; (4) a celecoxib group, receiving 10 mg/kg of celecoxib every other day for 8 weeks.

To ensure proper comparison, a control group was included in the study. Mice in the SHAM group underwent sham surgery and were treated with normal saline via oral gavage as the vehicle control.

### 4.3. Micro-CT Imaging and Bone Morphology Analysis

The skeletal and cartilaginous architecture of small-animal knee joints was investigated using a high-resolution in vivo micro-CT scanner (CT80X; Olympus, Tokyo, Japan). Scanning was performed at a resolution of 9 μm per pixel, with an exposure time of 900 ms, a tube voltage of 50 kV, and a tube current of 500 μA. Raw image data were reconstructed in three dimensions using ctVOX software (ctVOX 3.0; Olympus). Quantitative analyses of the reconstructed volumes were then carried out with CTAn (CTAN 2.0; Olympus) and DataViewer (DataViewer 1.5; Olympus). The region of interest was centered on the fracture zone around the knee joint, with particular emphasis on the interval between the tibial subchondral plate and the tibial articular cartilage surface. Reported quantitative metrics comprised bone volume fraction (BV/TV), trabecular separation (Tb.Sp), bone mineral density (BMD), and trabecular thickness (Tb.Th).

### 4.4. Histopathological Assessment

Following euthanasia by CO_2_, the whole hindlimb knee joint specimens were dissected and collected. The specimens were fixed in 4% paraformaldehyde for 24 h, and then decalcified in EDTA solution using a slow protocol, with the decalcification medium replaced every 48 h; the total decalcification time was approximately one month. After decalcification, the samples were dehydrated through a graded ethanol series and processed for sagittal paraffin embedding according to standard procedures. Tissue blocks were sectioned continuously at 5 μm thickness. The sections were stained with hematoxylin and eosin (H&E) and Masson’s trichrome to assess cartilage damage severity and collagen fiber deposition, respectively. Representative images were captured using the microscope system ZEISS Axio Vert.A1 microscope (Carl Zeiss, Jena, Germany)

Cartilage degeneration was evaluated using the OARSI scoring system following the recommendations of Glasson et al. (2010) for murine models [51]. Three standardized sagittal sections from each knee joint were independently evaluated, and the cumulative score was calculated to yield a total OARSI score ranging from 0 to 18 per mouse.

Histopathological changes were assessed using a modified Mankin scoring system based on the original criteria described by Mankin et al. (1971) (score range 0–14 per section) [52]. All sections were evaluated in a double-blinded manner by two independent observers, and the mean score was used for statistical analysis. Following OARSI whole-joint recommendations, cartilage, subchondral bone, and synovium were evaluated to ensure an integrated histopathological assessment.

### 4.5. UPLC–MS/MS Analysis of BLQYF

BLQYF was prepared according to a standardized formulation, and the dried materials were pulverized and extracted with 70% methanol by ultrasonic extraction at room temperature. After centrifugation at 12,000× *g* for 10 min, the supernatants were filtered through 0.22 μm membranes prior to analysis. UPLC–MS/MS analysis was performed using an Agilent 1290 Infinity II ultra-performance liquid chromatography system coupled with an Agilent 6495D Triple Quadrupole mass spectrometer, equipped with a C18 column (2.1 × 100 mm, 1.7 μm), with the column temperature maintained at 30 °C. The mobile phase consisted of 0.1% formic acid in water (solvent A) and acetonitrile (solvent B), delivered at a flow rate of 0.3 mL min^−1^ under gradient elution, with an injection volume of 2 μL. Mass spectrometric detection was conducted on a triple quadrupole mass spectrometer with an electrospray ionization source operating in both positive and negative ion modes, and data were acquired in multiple reaction monitoring mode. Authentic reference standards of bavachinin, hederagenin, myricanone, kaempferol, β-sitosterol, cyclo(Gly–Pro), and ursolic acid (purity ≥ 98%) were used for compound identification and quantification. Data acquisition and qualitative analysis were performed using Agilent MassHunter Workstation Qualitative Analysis software (V12.0, Agilent Technologies, Santa Clara, CA, USA). Calibration curves were generated by plotting peak areas against standard concentrations, all of which showed good linearity (R^2^ > 0.99). The contents of individual compounds in BLQYF were calculated from the corresponding calibration curves and expressed as μg g^−1^ of dry extract. All analyses were performed in triplicate.

### 4.6. Active Compound Screening of BLQYF

The chemical constituents of Bailong Qufeng Yao Formula (BLQYF) were retrieved from publicly available traditional Chinese medicine databases, including the Traditional Chinese Medicine Systems Pharmacology Database (TCMSP, https://www.tcmsp-e.com/load_intro.php?id=43 (accessed on 1 June 2025)) and relevant literature sources. To identify bioactive compounds with favorable pharmacokinetic properties, compounds were screened based on oral bioavailability (OB ≥ 30%) and drug-likeness (DL ≥ 0.18). Compounds not meeting these criteria but reported in the literature to possess biological or pharmacological activity were retained for further analysis.

### 4.7. Target Prediction of BLQYF-Related Compounds

Potential molecular targets of the screened BLQYF active compounds were predicted using the Traditional Chinese Medicine Systems Pharmacology Database (TCMSP, https://www.tcmsp-e.com/load_intro.php?id=43, accessed on 1 June 2025) and SwissTargetPrediction (http://www.swisstargetprediction.ch/, accessed on 1 June 2025). All predicted targets were standardized by mapping to official gene symbols using the UniProt database (https://www.uniprot.org/, accessed on 1 June 2025), with the species restricted to *Homo sapiens*. Duplicate targets were removed to generate a non-redundant BLQYF target set.

### 4.8. Identification of PTOA-Related Targets

Post-traumatic osteoarthritis (PTOA)-associated genes were collected from disease-related databases, including GeneCards (https://www.genecards.org/, accessed on 1 June 2025), GEO (https://www.ncbi.nlm.nih.gov/geo/, accessed on 1 June 2025), and DisGeNET (https://www.disgenet.org/, accessed on 1 June 2025), using the keyword “post-traumatic osteoarthritis.” Targets with relevance scores above the median value in GeneCards were selected to improve disease specificity. All retrieved disease-related targets were standardized and merged after removing duplicates.

### 4.9. Intersection Target Analysis

The overlapping targets between BLQYF-related targets and PTOA-associated targets were identified using Venn diagram analysis. These intersecting targets were considered potential therapeutic targets through which BLQYF may exert its effects on PTOA and were subjected to subsequent network and pathway analyses.

### 4.10. Protein–Protein Interaction (PPI) Network Construction and Analysis

The intersecting targets were imported into the STRING database (https://string-db.org/, accessed on 1 June 2025), with the species limited to Homo sapiens.. A minimum required interaction score of 0.4 (medium confidence) was applied to construct the protein–protein interaction (PPI) network. The resulting PPI network was visualized and analyzed using Cytoscape software (version 3.9.1). Key targets were identified based on topological parameters, including degree, betweenness centrality, and closeness centrality.

### 4.11. GO Functional Annotation and Pathway Enrichment Analysis

Gene Ontology (GO) functional annotation and Kyoto Encyclopedia of Genes and Genomes (KEGG) pathway enrichment analyses were performed for the intersecting targets using the Database for Annotation, Visualization and Integrated Discovery (DAVID) and/or the Metascape platform. Enrichment results were considered statistically significant when *p* < 0.05. The enriched biological processes and signaling pathways were visualized to elucidate the potential biological functions and molecular mechanisms underlying the effects of BLQYF in PTOA.

### 4.12. Network Construction and Visualization

An integrated “compound–target–pathway” network was constructed using Cytoscape 3.9.1 (Cytoscape Consortium, San Diego, CA, USA) to illustrate the multi-component, multi-target, and multi-pathway regulatory characteristics of BLQYF in PTOA. Nodes represent compounds, targets, or pathways, while edges indicate their interactions. Network topology analysis was conducted to identify key compounds and core targets within the network.

### 4.13. Molecular Docking

Molecular docking was conducted to evaluate the interactions between the main active compounds (Bavachinin, Hederagenin, and Myricanone) and target proteins (FN1, MMP3, and TGFB1). The crystal structures of FN1 (PDB ID: 5J6Z), MMP3 (PDB ID: 2JT5), and TGFB1 (PDB ID: 1KLC) were obtained from the Protein Data Bank. Water molecules and co-crystallized ligands were removed, polar hydrogens were added, and Gasteiger charges were assigned using AutoDock Vina 1.2.0 (The Scripps Research Institute, La Jolla, CA, USA) before conversion to PDBQT format. Ligand structures were retrieved from PubChem, energy-minimized with the MMFF94 force field, and prepared in PDBQT format. Docking simulations were performed using AutoDock Vina (version 1.1.2). The grid box was defined to cover the predicted binding region (approximately 30 × 30 × 30 Å), and for MMP3, the grid was centered on the native ligand-binding site. Exhaustiveness was set to 8. The optimal binding pose was selected based on the lowest predicted binding energy (kcal/mol) and visualized using PyMOL 2.5 (Schrödinger, LLC, New York, NY, USA). Docking results represent computational predictions and require further experimental validation.

### 4.14. Single-Cell RNA-Seq Data Acquisition and Preprocessing

Single-cell RNA sequencing data were obtained from the Gene Expression Omnibus (GEO) database (GSE216651) and processed using Seurat (v4.0). Low-quality cells and potential doublets were excluded based on gene count thresholds and mitochondrial gene expression. Data were log-normalized and scaled, and highly variable genes were selected for principal component analysis (PCA). Uniform manifold approximation and projection (UMAP) was applied for dimensionality reduction, followed by unsupervised clustering using a shared nearest neighbor (SNN) graph-based approach. Cell clusters were annotated according to established canonical marker genes.

Cell-type proportions were calculated and compared between healthy (H1) and osteoarthritic (O1) samples. To identify coordinated transcriptional programs, single-cell weighted gene co-expression network analysis (scRNA-seq WGCNA) was performed separately for H1 and O1 samples using normalized expression data. Co-expressed genes were grouped into modules by hierarchical clustering based on topological overlap. Module eigengenes were computed and projected onto the UMAP space to assess cell-type-associated module enrichment. The expression of representative module-associated genes was visualized using feature and violin plots.

Functional enrichment analysis of selected co-expression modules was conducted using Gene Ontology (GO) biological process annotation. Enriched terms with *p* < 0.05 were considered statistically significant.

### 4.15. Serum ELISA

At the indicated time points, whole blood was collected and serum was isolated by centrifugation. The serum concentrations of MMP3 (Cat# E-EL-M0626, Elabscience Biotechnology Co., Ltd., Wuhan, China), TGF-β (Cat# E-EL-M0051, Elabscience Biotechnology Co., Ltd., Wuhan, China), and FN1 (Cat# KE00039, Elabscience Biotechnology Co., Ltd., Wuhan, China) were quantified by ELISA using commercial kits according to the manufacturers’ instructions.

### 4.16. Isolation and Culture of Human Fibroblast-like Synoviocytes

Primary fibroblast-like synoviocytes (FLSs) were isolated from synovial tissues obtained from patients with post-traumatic osteoarthritis undergoing knee surgery. The study was approved by the Ethics Committee of Yangzhou Hospital of Traditional Chinese Medicine (Yangzhou, China) (Approval No. 2022-LS-24), and written informed consent was obtained from all patients prior to sample collection. Synovial tissues were enzymatically digested, and adherent cells were expanded in culture. FLS identity was confirmed based on spindle-shaped morphology and immunofluorescence staining for the mesenchymal marker vimentin.

### 4.17. Mesenchymal Stem Cell Culture and Cell Viability Assay

Mesenchymal stem cells (MSCs) were isolated from bone marrow samples obtained from patients undergoing total hip arthroplasty for non-osteoarthritic conditions. Cells were treated with BLQYF (0–80 μg/mL) for 24 h, and cell viability was assessed using a CCK-8 assay.

### 4.18. siRNA Transfection and qRT–PCR

Primary human fibroblast-like synoviocytes (FLSs) were transfected with *FN1*-targeting siRNA or a non-targeting control siRNA using a standard lipid-based transfection reagent. After transfection, cells were treated with BLQYF (5–20 μg/mL) for 24 h. Total RNA was extracted, reverse transcribed, and analyzed by qRT–PCR. Relative gene expression levels were normalized to housekeeping genes and calculated using the ΔΔCt method.

### 4.19. Ethical Approval

All animal experiments were conducted in accordance with the National Guidelines for the Care and Use of Laboratory Animals and were approved by the Experimental Animal Welfare and Ethics Committee of Yangzhou University (Approval No. 2022).

SPF-grade BALB/c mice were housed in a barrier facility under standard laboratory conditions (22 ± 2 °C, 50–60% humidity, 12 h light/dark cycle) with free access to food and water. All efforts were made to minimize animal suffering.

Human synovial tissues and bone marrow samples were obtained with informed consent from all participants. The human study protocol was approved by the Ethics Committee of Yangzhou University Affiliated Hospital (Yangzhou Hospital of Traditional Chinese Medicine) (Approval No. 2022-LS-24) and conducted in accordance with the Declaration of Helsinki.

### 4.20. Statistical Analysis

Data were analyzed using GraphPad Prism 9. Data normality was tested using the Shapiro–Wilk test. For comparisons between two groups, Student’s t-test was used, and for multiple group comparisons, one-way ANOVA followed by Tukey’s post hoc test was applied. The sample size for each group was determined based on a priori power analysis. Statistical significance was defined as *p* < 0.05, *p* < 0.01, *p* < 0.001, and *p* < 0.0001, with ‘ns’ indicating *p* ≥ 0.05 (non-significant differences).

## 5. Conclusions

BLQYF was well tolerated under the present experimental conditions, based on serum biochemical parameters and histopathological evaluation, and subchondral bone damage in a murine model of post-traumatic osteoarthritis, with therapeutic efficacy comparable to celecoxib.

Mechanistically, BLQYF modulates fibroblast-mediated inflammation and extracellular matrix remodeling through suppression of fibronectin 1 (FN1) and downstream inflammatory mediators. These findings support BLQYF as a promising disease-modifying therapeutic candidate for PTOA and identify the fibroblast–FN1 axis as a potential pharmacological target.

## Data Availability

The original contributions presented in this study are included in the article/Supplementary Material. Further inquiries can be directed to the corresponding authors.

## Supplementary Materials

The following supporting information can be downloaded at: https://www.mdpi.com/article/10.3390/ph19030500/s1, Figure S1: Evaluation of the safety of BLQYF on liver and kidney function.; Figure S2: Chemical structures and standard calibration curves of the six active components; Figure S3: Network pharmacology analysis and functional enrichment of potential targets; Figure S4: Identification of cell-type specific gene co-expression modules using single-cell data; Table S1: Individual OARSI scores assessed independently by two blinded observers; Table S2: Individual Modified Mankin scores assessed independently by two blinded observers.

## Abbreviations

PTOA	Post-Traumatic Osteoarthritis
BLQYF	Biluo Qianyuan Formula
FN1	Fibronectin 1
MMP3	Matrix Metalloproteinase 3
TGF-β	Transforming Growth Factor Beta
NSAIDs	Non-Steroidal Anti-Inflammatory Drugs
DMOADs	Disease-Modifying Osteoarthritis Drugs
CT	Computed Tomography
OARSI	Osteoarthritis Research Society International
scRNA-seq	Single-Cell RNA Sequencing
UMAP	Uniform Manifold Approximation and Projection
PPI	Protein–Protein Interaction
GO	Gene Ontology
KEGG	Kyoto Encyclopedia of Genes and Genomes
H&E	Hematoxylin and Eosin
UPLC–MS/MS	Ultra Performance Liquid Chromatography–Tandem Mass Spectrometry
