# Supplementary material for: Biluo Qianyuan Formula Ameliorates Post-Traumatic Osteoarthritis by Suppressing FN1-Mediated Synovial Inflammation and Restoring Joint Homeostasis
Source: Pharmaceuticals (Basel). 2026 Mar 18;19(3):500. doi: 10.3390/ph19030500 (PMC13028745; doi:10.3390/ph19030500)
Supplement: Supplementary file 1 [file pharmaceuticals-19-00500-s001.zip › pharmaceuticals-4146497-supplementary_figures.pdf]

Supporting Information for

Biluo Qianyuan Formula ameliorates post-traumatic osteoarthritis by suppressing FN1-mediated synovial inflammation and restoring joint homeostasis

Yinqiu Wu [1, 2, 3], Guangran Hu [1, 2, 3], Shengzhe Zhang [1, 2], Guilan Jin [3,*] and Hua Dai [1, 2,*]

1 School of Basic Medical Sciences & School of Public Health, Faculty of Medicine, Yangzhou University, Yangzhou 225001, China

2 The Key Laboratory of the Jiangsu Higher Education Institutions for Nucleic Acid & Cell Fate Regulation, Yangzhou University, Yangzhou 225001, China

3 School of Basic Medical Sciences, Nanjing University of Chinese Medicine,

* Correspondence: molianbing@126.com (G.J.); daihua@yzu.edu.cn (H.D.)

This PDF file includes:

Figures S1 to S4.

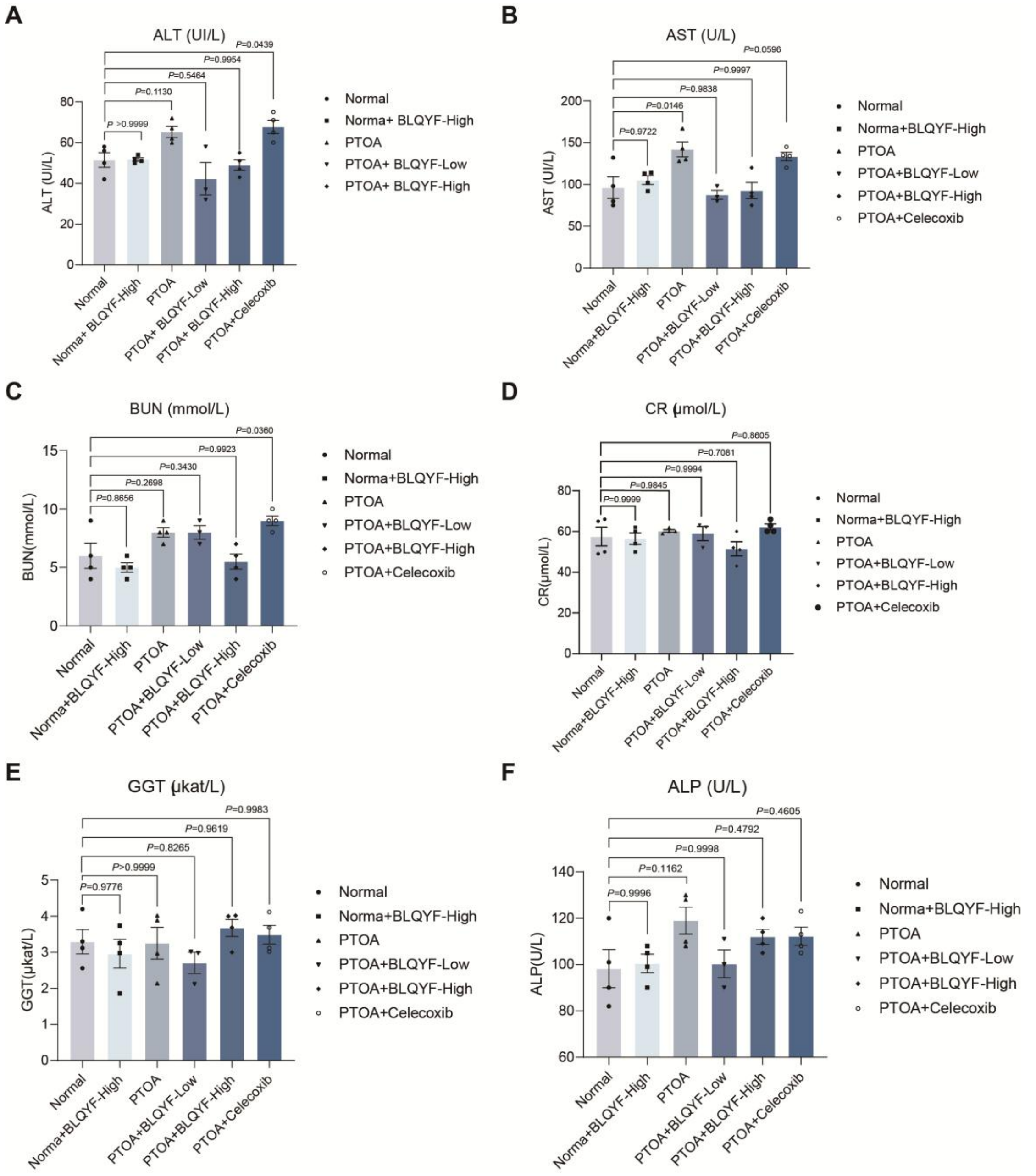
A
ALT (UI/L)
P=0.0439
P=0.9954
P=0.5464
P=0.1130
P >0.9999
ALT (UI/L)
Normal
Norma+ BLQYF-High
PTOA
PTOA+ BLQYF-Low
PTOA+ BLQYF-High
PTOA+Celecoxib
B
AST (U/L)
P=0.0596
P=0.9997
P=0.9838
P=0.0146
P=0.9722
AST (UI/L)
Normal
Norma+BLQYF-High
PTOA
PTOA+BLQYF-Low
PTOA+BLQYF-High
PTOA+Celecoxib
C
BUN (mmol/L)
P=0.0360
P=0.9923
P=0.3430
P=0.2698
P=0.8656
BUN(mmol/L)
Normal
Norma+BLQYF-High
PTOA
PTOA+BLQYF-Low
PTOA+BLQYF-High
PTOA+Celecoxib
D
CR (μmol/L)
P=0.8605
P=0.7081
P=0.9994
P=0.9845
P=0.9999
CR(μmol/L)
Normal
Norma+BLQYF-High
PTOA
PTOA+BLQYF-Low
PTOA+BLQYF-High
PTOA+Celecoxib
E
GGT (μkat/L)
P=0.9983
P=0.9619
P=0.8265
P>0.9999
P=0.9776
GGT(μkat/L)
Normal
Norma+BLQYF-High
PTOA
PTOA+BLQYF-Low
PTOA+BLQYF-High
PTOA+Celecoxib
F
ALP (U/L)
P=0.4605
P=0.4792
P=0.9998
P=0.1162
P=0.9996
ALP(U/L)
Normal
Norma+BLQYF-High
PTOA
PTOA+BLQYF-Low
PTOA+BLQYF-High
PTOA+Celecoxib

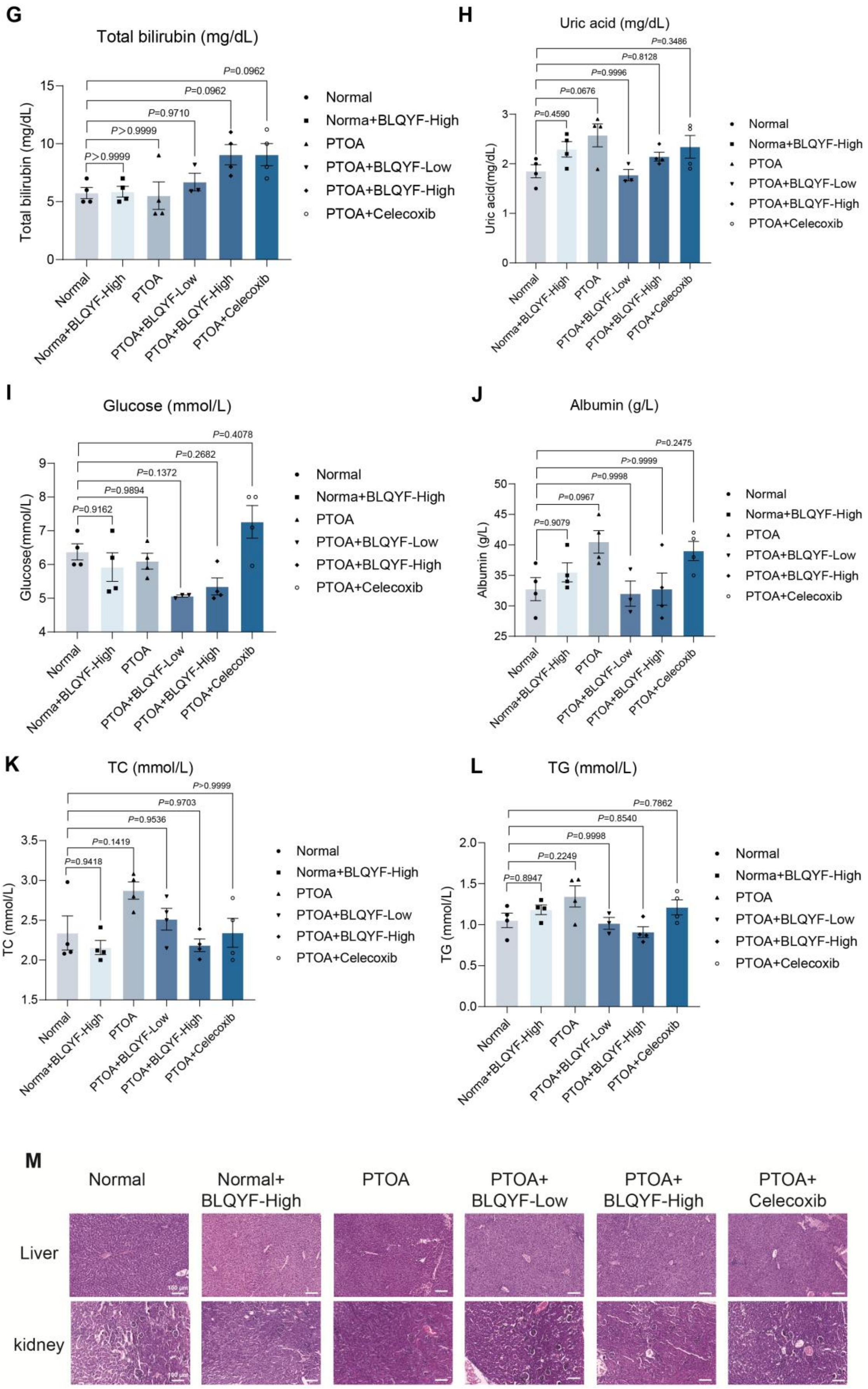
G
Total bilirubin (mg/dL)
Total bilirubin (mg/dL)
P>0.9999
P>0.9999
P=0.9710
P=0.0962
P=0.0962
Normal
Norma+BLQYF-High
PTOA
PTOA+BLQYF-Low
PTOA+BLQYF-High
PTOA+Celecoxib
H
Uric acid (mg/dL)
Uric acid(mg/dL)
P=0.4590
P=0.0676
P=0.9996
P=0.8128
P=0.3486
Normal
Norma+BLQYF-High
PTOA
PTOA+BLQYF-Low
PTOA+BLQYF-High
PTOA+Celecoxib
I
Glucose (mmol/L)
Glucose(mmol/L)
P=0.9162
P=0.9894
P=0.1372
P=0.2682
P=0.4078
Normal
Norma+BLQYF-High
PTOA
PTOA+BLQYF-Low
PTOA+BLQYF-High
PTOA+Celecoxib
J
Albumin (g/L)
Albumin (g/L)
P=0.9079
P=0.0967
P=0.9998
P>0.9999
P=0.2475
Normal
Norma+BLQYF-High
PTOA
PTOA+BLQYF-Low
PTOA+BLQYF-High
PTOA+Celecoxib
K
TC (mmol/L)
TC (mmol/L)
P=0.9418
P=0.1419
P=0.9536
P=0.9703
P>0.9999
Normal
Norma+BLQYF-High
PTOA
PTOA+BLQYF-Low
PTOA+BLQYF-High
PTOA+Celecoxib
L
TG (mmol/L)
TG (mmol/L)
P=0.8947
P=0.2249
P=0.9998
P=0.8540
P=0.7862
Normal
Norma+BLQYF-High
PTOA
PTOA+BLQYF-Low
PTOA+BLQYF-High
PTOA+Celecoxib
M
Normal
Normal+ BLQYF-High
PTOA
PTOA+ BLQYF-Low
PTOA+ BLQYF-High
PTOA+ Celecoxib
Liver
kidney

Supplementary Figure S1

Evaluation of the safety of BLQYF on liver and kidney function.

Serum biochemical parameters were measured to assess potential hepatotoxicity and nephrotoxicity in different groups (Normal, Normal+BLQYF-High, PTOA, PTOA+BLQYF-Low, PTOA+BLQYF-High, and PTOA+Celecoxib). (A–F) Liver function markers including ALT, AST, GGT, ALP, total bilirubin, and albumin. (C–D, H) Renal function indices including BUN, creatinine (CR), and uric acid. (I–L) Glucose and lipid profiles (glucose, TC, and TG). (M) Representative H&E staining of liver and kidney tissues. Data are presented as mean ± SEM.

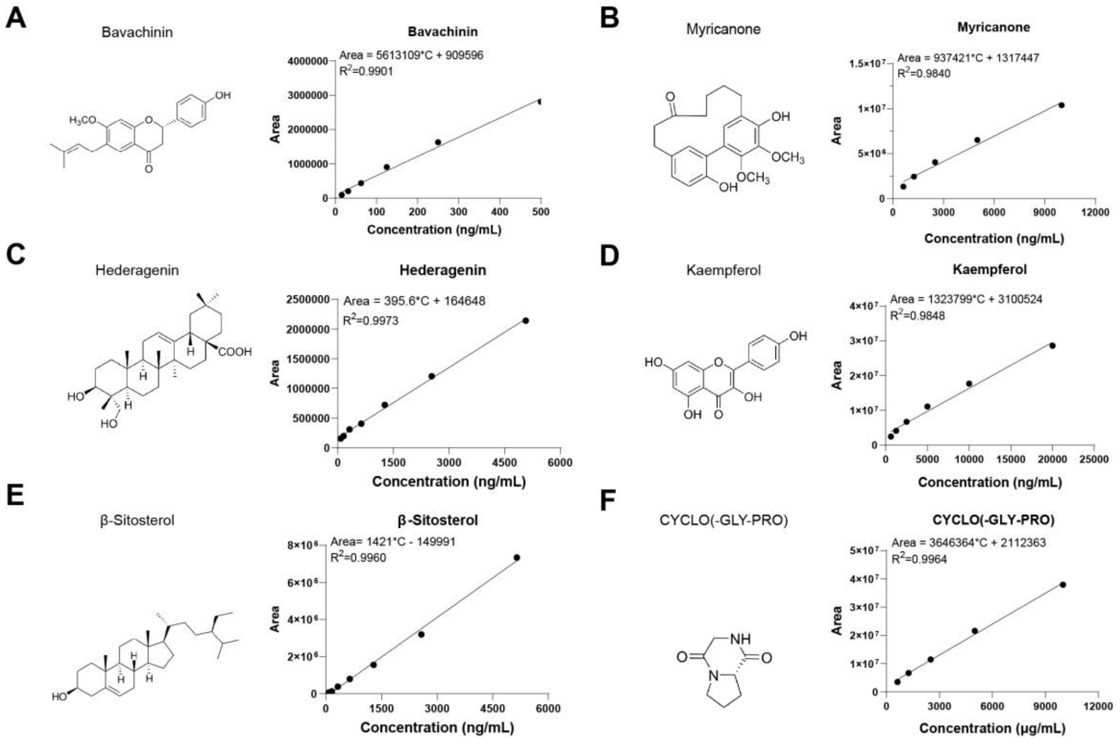

Supplementary Figure S2

Chemical structures and standard calibration curves of the six active components.

The panels display the chemical structure (left) and the linear regression analysis of peak area versus concentration (right) for: (A) Bavachinin; (B) Myricanone; (C) Hederagenin; (D) Kaempferol; (E) β-Sitosterol; and (F) CYCLO(-GLY-PRO). The

linear regression equation and the correlation coefficient R^2 are presented in each graph, indicating the linearity of the quantification method.

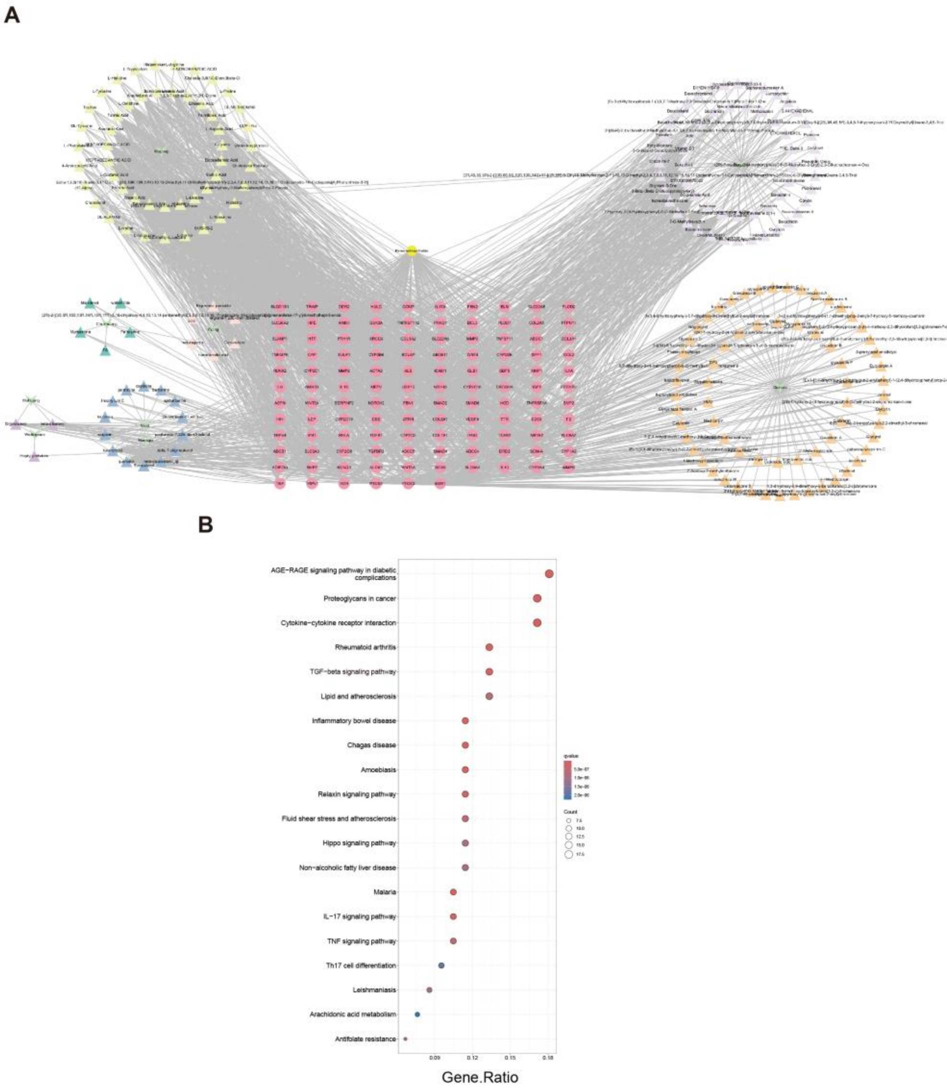

Supplementary Figure S3

Network pharmacology analysis and functional enrichment of potential targets.

(A) Construction of the Compound-Target interaction network. The peripheral clusters of nodes represent the bioactive components, while the central red nodes represent the intersecting potential gene targets associated with the disease. This visualizes the multi-component and multi-target mechanisms. (B) Bubble chart of the Kyoto

Encyclopedia of Genes and Genomes (KEGG) pathway enrichment analysis. The top 20 significantly enriched signaling pathways are displayed (including AGE-RAGE signaling pathway, Rheumatoid arthritis, etc.). The x-axis represents the Gene Ratio; the size of the bubbles indicates the number of genes enriched in the pathway (Count); and the color gradient (from blue to red) indicates the statistical significance (q-value), with redder colors denoting higher significance.

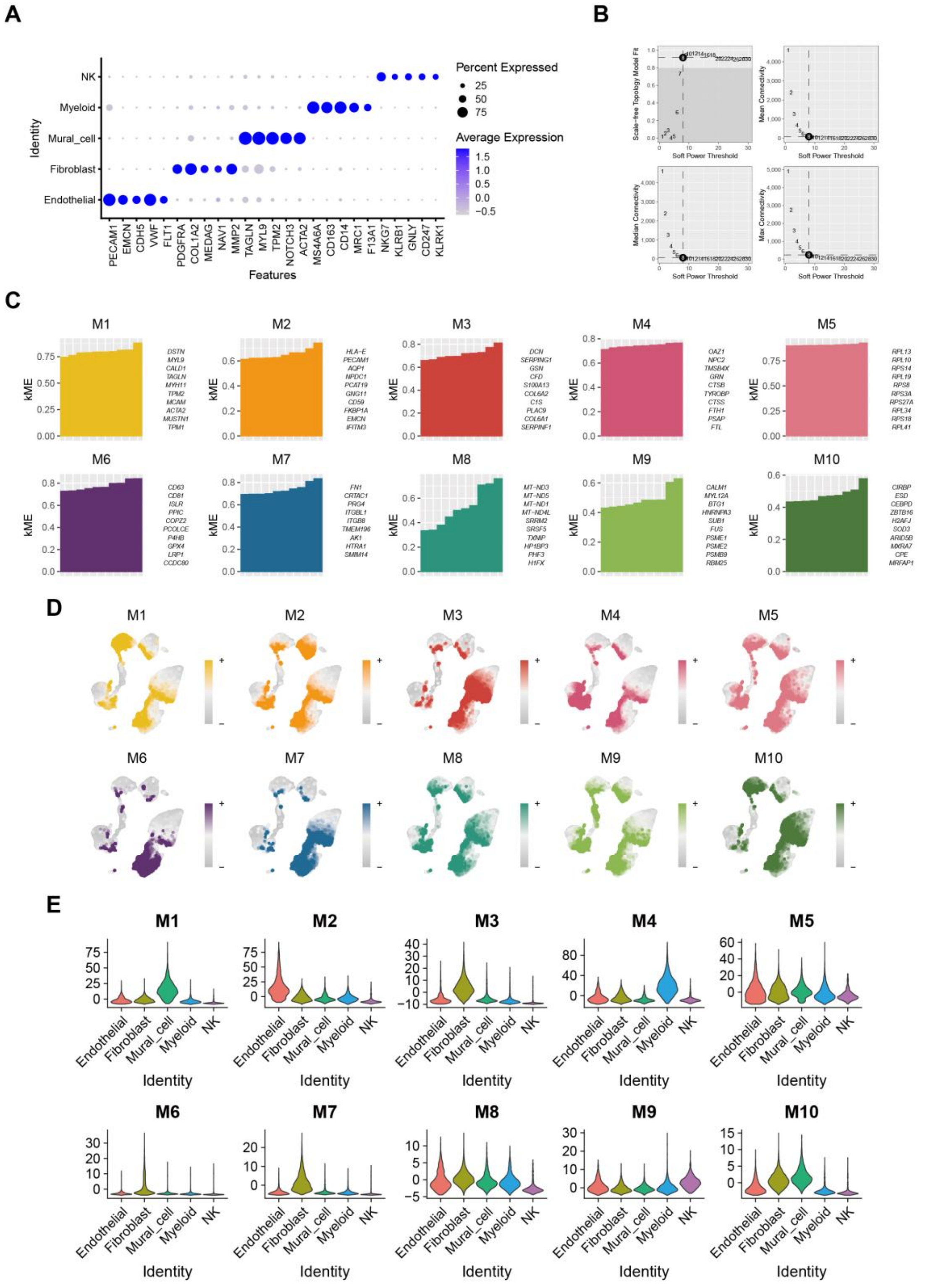

Supplementary Figure S4

Identification of cell-type specific gene co-expression modules using single-cell data.

(A) Dot plot showing the expression of canonical marker genes used to annotate five major cell types: Endothelial, Fibroblast, Mural cells, Myeloid, and NK cells. Dot size

represents the percentage of cells expressing the gene, and color intensity indicates the average expression level. (B) Analysis of network topology for various soft-thresholding powers to determine the optimal parameter for constructing a scale-free network (WGCNA). (C) Visualization of the top hub genes for each of the ten identified gene modules (M1–M10), ranked by their intramodular connectivity (kME). (D) UMAP feature plots displaying the spatial distribution of module activity scores across the single-cell landscape. (E) Violin plots showing the distribution of module scores across different cell identities, highlighting the cell-type specificity of each module (e.g., M1/M2 in endothelial cells, M3/M4 in fibroblasts).
