# Supplementary material for: Biluo Qianyuan Formula Ameliorates Post-Traumatic Osteoarthritis by Suppressing FN1-Mediated Synovial Inflammation and Restoring Joint Homeostasis
Source: Pharmaceuticals (Basel). 2026 Mar 18;19(3):500. doi: 10.3390/ph19030500 (PMC13028745; doi:10.3390/ph19030500)
Supplement: Supplementary file 1 [file pharmaceuticals-19-00500-s001.zip › pharmaceuticals-4146497-supplementary_tables.pdf]

Supplementary Table S1

Individual OARSI scores assessed independently by two blinded observers

Group	Mouse ID	Observer 1	Observer 2	Mean score per mouse
SHAM	1	1	0	0.5
SHAM	2	1	1	1.0
SHAM	3	0	1	0.5
SHAM	4	1	1	1.0
PTOA	1	16	17	16.5
PTOA	2	15	14	14.5
PTOA	3	18	17	17.5
PTOA	4	16	17	16.5
PTOA + BLQYF-Low	1	12	11	11.5
PTOA + BLQYF-Low	2	10	11	10.5
PTOA + BLQYF-Low	3	8	8	8.0
PTOA + BLQYF-High	1	6	5	5.5
PTOA + BLQYF-High	2	5	5	5.0
PTOA + BLQYF-High	3	4	4	4.0
PTOA + BLQYF-High	4	3	4	3.5

Group	Mouse ID	Observer 1	Observer 2	Mean score per mouse
PTOA + Celecoxib	1	2	3	2.5
PTOA + Celecoxib	2	5	4	4.5
PTOA + Celecoxib	3	4	4	4.0
PTOA + Celecoxib	4	4	4	4.0

All sections were evaluated independently by two observers blinded to treatment allocation. The mean score per animal was used for statistical analysis.

Supplementary Table S2

Individual Modified Mankin scores assessed independently by two blinded observers

Group	Mouse ID	Observer 1	Observer 2	Mean score per mouse
SHAM	1	1	0	0.5
SHAM	2	0	1	0.5
SHAM	3	0	0	0.0
SHAM	4	0	0	0.0
PTOA	1	14	12	13.0
PTOA	2	13	10	11.5
PTOA	3	10	9	9.5
PTOA	4	14	12	13.0
PTOA + BLQYF-Low	1	5	7	6.0
PTOA + BLQYF-Low	2	8	6	7.0
PTOA + BLQYF-Low	3	10	10	10.0
PTOA + BLQYF-High	1	7	5	6.0
PTOA + BLQYF-High	2	5	5	5.0
PTOA + BLQYF-High	3	2	4	3.0
PTOA + BLQYF-High	4	3	3	3.0

Group	Mouse ID	Observer 1	Observer 2	Mean score per mouse
PTOA + Celecoxib	1	4	5	4.5
PTOA + Celecoxib	2	6	5	5.5
PTOA + Celecoxib	3	4	3	3.5
PTOA + Celecoxib	4	3	4	3.5

All sections were evaluated independently by two observers blinded to treatment allocation. The mean score per animal was used for statistical analysis.
